# Missed Opportunities of Flu Vaccination in Italian Target Categories: Insights from the Online EPICOVID 19 Survey

**DOI:** 10.3390/vaccines8040669

**Published:** 2020-11-09

**Authors:** Andrea Giacomelli, Massimo Galli, Stefania Maggi, Gabriele Pagani, Raffaele Antonelli Incalzi, Claudio Pedone, Mauro Di Bari, Marianna Noale, Caterina Trevisan, Fabrizio Bianchi, Marcello Tavio, Massimo Andreoni, Claudio Mastroianni, Aleksandra Sojic, Federica Prinelli, Fulvio Adorni

**Affiliations:** 1 Department of Biomedical and Clinical Sciences L. Sacco, Università di Milano, Via G.B. Grassi 74, 20157 Milan, Italy; massimo.galli@unimi.it (M.G.); gabriele.pagani@unimi.it (G.P.); 2 National Research Council, Institute of Neuroscience, Aging branch, Via Vincenzo Maria Gallucci 16, 35128 Padova, Italy; stefania.maggi@in.cnr.it (S.M.); marianna.noale@in.cnr.it (M.N.); caterina.trevisan.5@studenti.unipd.it (C.T.); 3 Unit of Geriatrics, Department of Medicine, Biomedical Campus of Rome, via Alvaro del Portillo, 21, 00128 Rome, Italy; r.antonelli@unicampus.it (R.A.I.); claudio.pedone@gmail.com (C.P.); 4 Geriatric Intensive Care Medicine, University of Florence and Azienda Ospedaliero-Universitaria Careggi, Viale Peraccini 18, 50139 Florence, Italy; mauro.dibari@unifi.it; 5 Geriatric Unit, Department of Medicine (DIMED), University of Padova, Via Giustiniani 2, 35128 Padova, Italy; 6 National Research Council, Institute of Clinical Physiology, Via G. Moruzzi 1, 56124 Pisa, Italy; fabriepi@ifc.cnr.it; 7 Division of Infectious Diseases, Azienda Ospedaliero Universitaria Ospedali Riuniti, Via Conca, 71, 60126 Ancona, Italy; marcello.tavio@ospedaliriuniti.marche.it; 8 Infectious Diseases Clinic, Department of System Medicine, Tor Vergata University of Rome, Via Cracovia, 50, 00133 Rome, Italy; andreoni@uniroma2.it; 9 Public Health and Infectious Disease Department, “Sapienza” University, Piazzale Aldo Moro, 5, 00185 Rome, Italy; claudio.mastroianni@uniroma1.it; 10 National Research Council, Institute of Biomedical Technologies, Via Fratelli Cervi 93, 20090 Segrate, Italy; aleksandra.sojic@itb.cnr.it (A.S.); federica.prinelli@itb.cnr.it (F.P.); fulvio.adorni@itb.cnr.it (F.A.)

**Keywords:** SARS-CoV-2, COVID-19, influenza, vaccine, elderly, Italy

## Abstract

We aimed to assess the reported rate of flu vaccination in the 2019/2020 season for respondents to the Italian nationwide online EPICOVID 19 survey. A national convenience sample of volunteers aged 18 or older was assessed between 13 April and 2 June 2020. Flu vaccine rates were calculated for all classes of age. The association between the independent variables and the flu vaccine was assessed by applying a multivariable binary logistic regression model. Of the 198,822 respondents, 41,818 (21.0%) reported having received a flu vaccination shot during the last influenza season. In particular, 15,009 (53.4%) subjects aged 65 years or older received a flu vaccination shot. Being 65 years aged or older (Adjusted Odds Ratios (aOR) 3.06, 95% Confidence Interval (CI) 2.92–3.20) and having a high education level (aOR 1.34. 95%CI 1.28–1.41) were independently associated to flu vaccination. Heart and lung diseases were the morbidities associated with the higher odds of being vaccinated (aOR 1.97 (95%CI 1.86–2.09) and aOR 1.92 (95%CI 1.84–2.01), respectively). Nursing home residents aged ≥ 65 years showed lower odds of being vaccinated (aOR 0.39 (95%CI 0.28–0.54)). Our data indicate the need for an urgent public heath effort to fill the gap of missed vaccination opportunities reported in the past flu seasons.

## 1. Introduction

Influenza is an acute viral infection of the respiratory tract, whose clinical manifestations range from mild upper respiratory tract symptoms to life-threating complications requiring hospitalization. It is estimated that every year flu can cause worldwide high morbidity and mortality epidemics with approximately 3 to 5 million cases and between 250,000 and 500,000 deaths [1]. In the northern hemisphere, influenza viruses circulating during fall and winter seasons cause epidemic outbreaks with an important burden of morbidity and a remarkable mortality rate. In Italy, the cost for the national healthcare system has been estimated at an average of USD 1.4 billion for each season between 1999 and 2008 [2].

The new pandemic caused by Severe Acute Respiratory Syndrome-Coronavirus-2 (SARS-CoV-2) has rapidly spread from China in late December 2019 and north of Italy in late February 2020 [3]. SARS-CoV-2 infection causes a severe respiratory syndrome which could lead to intensive care assistance and to death [4,5]. The dramatic impact of the SARS-CoV-2 pandemic on the Italian national healthcare system led the national authorities to impose a national lockdown from March 9 to May 18, 2020. After a period of apparent control in summer months, the current Italian epidemiological situation is evolving with a slight daily increment of the number of cases and a progressive increment of hospitalized patients [6]. Consequently, non-pharmacological interventions, such as the face mask combined with social distancing, are still enforced.

The new challenge in the management of the pandemic will appear in the 2020/2021 flu season, when the co-occurrence of influenza and flu-like illnesses may overcome the diagnostic capacity of our system with regard to the capability for molecularly testing all subjects with respiratory symptoms. Moreover, the clinical impact of subsequent or concomitant influenza and SARS-CoV-2 infections is still unknown, especially in the elderly populations and in patients with comorbidities.

To date, no effective treatment has been demonstrated to radically change the natural history of SARS-CoV-2 infection [7], and several vaccination strategies are still under development [8]; on the other hand, flu vaccination is able to decrease both the incidence rates of influenza and to moderate the severity of the disease in case of infection. Moreover, a remarkable decrease in morbidity for pneumonia, respiratory, and cardiovascular complications and consequent risk of hospitalization and death has been consistently reported in the literature [9,10,11,12]. The reduced risk of hospitalization is particularly important considering the significant amount of hospital resources that are required for the management of the non-negligible percentage of COVID-19 patients who required hospitalization and intensive care assistance [4,5]. In the end, influenza vaccinations are not only safe, with few adverse events related to its administration, but they are also proven to be effective in children above 6 months of age, people with comorbid conditions, and also in the elderly population [9,10,13].

Despite national and international recommendations for routine flu vaccination, vaccine uptake in Italy remains low in the general population as well as in the high-risk groups. Our aim was to assess the reported rate of flu vaccination in the 2019/2020 season in respondents to the nationwide online EPICOVID 19 survey [14] and to identify factors associated with the uptake of flu vaccination, in order to address potential gaps to be filled in the public health system.

## 2. Materials and Methods

### 2.1. Study Design and Setting

The study design was described elsewhere [14,15], and the study was registered (ClinicalTrials.gov NCT04471701). In brief, EPICOVID19 is an Italian cross-sectional, web-based survey that was initiated in April 2020. A national convenience sample of volunteers aged 18 or older with access to internet and able to give informed consent was assessed. The participants were recruited via social media (Facebook, Twitter, Instagram, WhatsApp), press releases, internet pages, local radio, TV stations, and institutional websites; the European Commission’s open-source official EUSurvey management tool was used to collect the data (https://epicovid19.itb.cnr.it/). The geographical coverage [14] such as the representativeness of the survey according to the Italian official demographic data [15] was described elsewhere.

### 2.2. Data Collection and Variables

For the purposes of this study, we analyzed all the survey responses collected between 13th April and 2nd June 2020. The socio-demographic information included sex (male vs. female, furtherly classified as pregnant or not), age (18–24, 25–29 up to 90+ and further categorized as <65 or ≥ 65 years of age), educational level (primary school or less = Low; middle or high school = Medium; and university degree or post-graduate = High), and occupational status (unemployed, employed, retired, student, and other). Work categories included in the national guidelines for flu vaccine were generated (health professionals, armed forces, agricultural, forestry, and fisher workers). Two ad hoc dummy variables were also created for categorizing residents in nursing homes (yes or no) and for respondents living with an “at risk cohabitant” according to the 2017–2019 Italian national vaccination plan (yes or no) [16]. Chronic conditions reported were lung diseases, heart diseases, metabolic disorders, renal diseases, oncological diseases, diseases of the immune system, liver diseases, and pre-planned major surgical procedures. A new variable was created by summing the chronic conditions referred by participants (categorized as none, 1, 2, or ≥3 comorbidities). Information about autonomy in carrying out daily activities and self-rated health (very bad, bad, adequate, good or very good) was also available. The participants were also asked if they had received an influenza vaccination during the previous autumn/winter season.

### 2.3. Study Population and Group Definition

Out of the 207,341 respondents, the study population consisted of 198,822 participants who provided informed consent, and for whom the data were fully available for the analysis. For the aim of this study, participants were categorized as vaccinated or not vaccinated for flu during the 2019/2020 season.

### 2.4. Statistical Analysis

Descriptive analyses were performed to show the numerical distributions, overall and by flu vaccine status, of all the variables that at a later stage were included as independent in the models applied for statistical inference. Continuous variables were expressed as mean (Standard Deviation (SD)), whereas categorical and ordinal ones were included as count and percentages. Flu vaccine rates were calculated for all classes of age and for all Italian regions, in this latter case also stratifying by sex (male vs. female) and age (<65 vs. ≥65 years). The association between the independent variables and the flu vaccine (0 = not reported, 1 = reported) was assessed by applying a multivariable binary logistic regression model. Adjusted Odds Ratios (aOR), together with their 95% Confidence Intervals (CI), were estimated for each independent variable, with a p-level of statistical significance set at 0.05, two tails. The same regression model was used when restricting the population analyzed to older people (≥65 years). All the statistical analyses were carried out using SPSS version 25 (IBM Corp.: Armonk, NY, USA) and STATA version 15.0 (StataCorp LLC: College Station, TX, USA).

### 2.5. Ethics and Consent to Participate

The Ethics Committee of the National Institute for Infectious Diseases (Italian: Istituto Nazionale per le Malattie Infettive I.R.C.C.S. Lazzaro Spallanzani) approved the EPICOVID19 study protocol (Protocol No. 70, 12/4/2020). The participants were requested to give their informed consent when they first accessed the online platform. The study was carried out in accordance with the principles of the Declaration of Helsinki.

All data were handled and stored in accordance with the European Union General Data Protection Regulation (EU GDPR) 2016/679; data transfer included encrypting/decrypting and password protection.

## 3. Results

Between April and June 2020, 198,822 participants filled the online EPICOVID 19 survey and 41,818 (21.0%) reported having received a flu vaccination shot during the last influenza season (2019/2020). Respondents were prevalently females (59.7%) and aged between 18 and 64 years (85.9%) with a mean age of 48 (SD ± 14.7 years) (Table 1).

Vaccination coverage progressively increased with age, from 11% between 18 and 24 years of age and peaking to 72.9% between 85 and 89 years of age (Supplementary Table S1).

More than half of the respondents (59.4%) had a high educational level, whereas 34.6% and 6% reported medium and low educational levels. More than seventy-five percent of respondents (149,168; 75%) reported no diseases, whereas the remaining 25% reported at least one clinical condition representing per se a flu vaccine indication. Vaccination coverage progressively increased up to 50.0% in those reporting three or more comorbidities. Among participants reporting at least one condition, those with heart disease showed the higher vaccination coverage (52.5%) followed by those reporting oncological diseases (40%) and metabolic disorders (39%), whereas only 34.6% of those with lung diseases were reportedly vaccinated for influenza. Among the professions with an indication for flu vaccination, healthcare workers were those with the higher coverage (35.7%). In the subgroup of healthcare professionals who reported a scientific profession as current employment (8621/14,736), thus excluding technicians and administrative personnel, the vaccination rate in these workers, who presumably were in direct contact with patients, was almost double (43.5% vs. 24.7%) respect to the remaining 6115 healthcare professionals.

**Figure 1:**
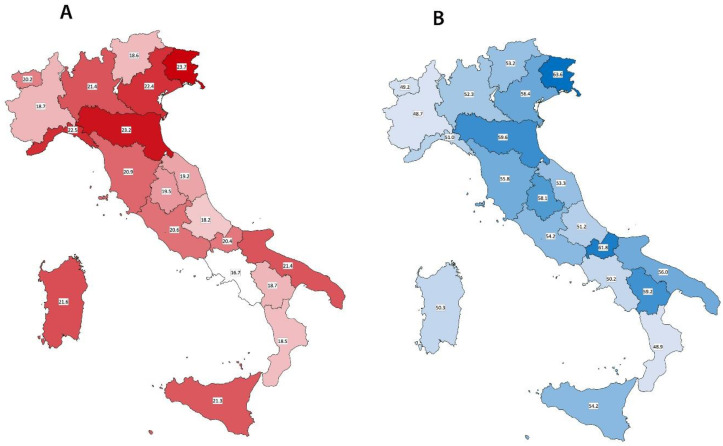
(**A**) Vaccination coverage according to the different Italian regions in respondents aged ≥ 18 years. (**B**), vaccination coverage according to the different Italian regions in respondents aged ≥ 65 years.

Vaccination coverage ranged widely across Italian regions from 18.2% in Abruzzo to 23.7% in Friuli-Venezia Giulia in the overall respondents (Figure 1A) and from 48.7% in Piemonte to 63.6% Friuli-Venezia Giulia in those aged ≥ 65 years (Figure 1B).

### Multivariable Model of Factors Associated to Flu Vaccination

**Figure 2:**
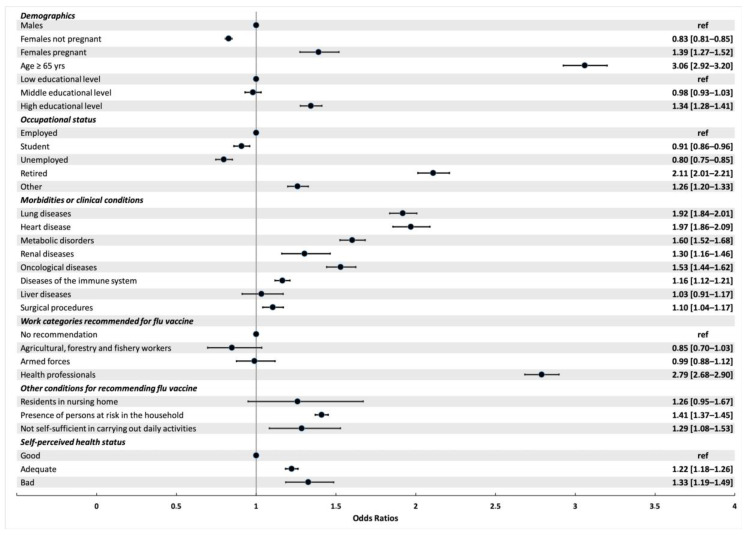
Multivariable model of factors associated to flu vaccination in the 2019/2020 season in respondents aged ≥ 18 years.

Figure 2 shows the multivariable model for subjects aged 18 years or older.

The multivariable model values stratified by age (18–64 years and ≥65 years) are reported in Table 2. When considering the whole respondents, being 65 years or older (aOR 3.06, 95% Confidence Interval (CI) 2.92–3.20) and having a high education level (aOR 1.34. 95%CI 1.28–1.41) were independently associated to flu vaccination. Conversely, those unemployed showed a reduced odd of being vaccinated (aOR 0.80, 95%CI 0.75–0.85).

Among comorbidities, heart and lung diseases were conditions associated with higher odds of being vaccinated, both in subjects aged 18–64 (aOR 2.15 (1.98 to 2.33) and aOR 1.95 (1.85 to 2.05), respectively) and in those aged above 65 years (aOR 1.54 (95%CI 1.42–1.67) and aOR 1.91 (95%CI 1.73–2.12), respectively). The odds of being vaccinated progressively increased with the number of reported morbidities with a more pronounced effect in those aged below 65 years (Supplementary Table S3).

Healthcare workers showed higher odds of being vaccinated (aOR 2.79 (95%CI 2.68–2.90)) when compared to a work position without vaccine recommendation.

Living in a nursing home and being unable to perform daily activities were both associated with lower odds of being vaccinated in those aged 65 years or older (aOR 0.39 (95%CI 0.28–0.54) and aOR 0.54 (95%CI 0.43–0.69), respectively).

## 4. Discussion

The web-based survey EPICOVID 19, which aimed to highlight different epidemiological and clinical aspects of COVID-19 during the first wave of the Italian epidemic, allowed the assessment of flu vaccination coverage in the 2019/2020 flu season in a large unselected sample of respondents. Moreover, we were able to identify predictors of flu vaccine uptake and highlight significant gaps in vaccination coverage in key populations such as in those residing in nursing homes and in those reporting specific medical conditions (i.e., liver diseases, kidney diseases, and diseases of the immune system).

Our findings showed an overall vaccination rate of 21% and a rate of 53.4% in those aged 65 years or older, which is clearly below the target recommended coverage. The available national data obtained from a surveillance system collecting data from the general practitioners since the 1999/2000 flu season report a progressive increase in the vaccination coverage until 2009/2010 for the overall population (19.6 per 100 inhabitants) and until 2008/2009 for those aged 65 years or older (66.3 per 100 inhabitants). A progressive decline was subsequently observed for those aged 65 years or older until 2014/2015 (48.5 per 100 inhabitants) followed by a slight progressive increase in the following years [17]. According to the official Italian Ministry of Health data about vaccination coverage in 2019/2020 flu season, the overall vaccination coverage in the whole Italian population was 16.7 over 100 inhabitants and 54.6 over 100 inhabitants in those aged 65 years or older, confirming the trend toward an increasing vaccination coverage [18]. Our data cannot be compared to the general estimates due to the absence of subjects aged 17 or younger in the surveyed population. However, the vaccination coverage observed in those aged 65 years or older appeared to be quite comparable to that reported by the official estimates (53.4% vs. 54.6%, respectively). Although slightly increasing in recent years, the percentage observed in our study is lower than the target coverage of 75% in those aged 65 years or older. These data are particularly worrisome because of the co-circulation of both influenza and SARS-CoV-2 viruses in the current northern hemisphere’s fall/winter season, which will pose major challenges due to the major diagnostic and clinical difficulties in managing upper and lower respiratory tract symptoms [19]. Based on these concerns, the Italian Ministry of Health recommends extending the vaccination coverage to the whole population during the 2020/2021 flu season, and the vaccine will be offered free of charge also for subject between 60 and 64 years of age [20]. Whether the free-of-charge administration in this age group will improve the uptake of the influenza vaccine remains to be demonstrated. However, it will help quantify the impact of the economical barrier to vaccination.

Recent findings support the need to increase vaccination coverage, considering, among the well-known health and economic advantages, also the observed association between flu vaccination and reduction in SARS-CoV-2 nasopharyngeal swab positivity [15] and lower COVID-19 severity and mortality [21,22,23]. Therefore, the impact of vaccinations should be assessed not only based on their ability to prevent the related infections but also on their broader impact on other communicable and non-communicable diseases occurring concurrently in the target population.

Our findings highlight a worrisome low vaccination coverage in subjects aged 60–64 years and 65–69 years (26.7% and 44.7%, respectively). The low vaccination coverage in this group could be partially explained by a self-perception of well-being and low risk perception [24]. Nevertheless, these age groups are at high risk of COVID-19 complications and mortality, as observed in the Italian COVID-19 patients; therefore, strategies to overcome their hesitancy toward vaccines and to increase vaccine accessibility and uptake are of paramount relevance [4,25].

Another interesting finding is that people with a higher education level seem to be better predisposed to vaccination when compared to a lower socio-economic level. If on the one hand, low education level is a known general barrier to vaccines [26], on the other, our sample of generally higher educational level compared to the general population reported overall a low uptake, meaning that multiple factors beyond health literacy and access to healthcare services need to be addressed. Moreover, the finding of a higher coverage among those with comorbidities suggests an attitude to consider the vaccination as a relevant intervention mainly for individuals at high risk rather than a meaningful intervention for public health promotion [27].

Our results show that patients with heart and lung diseases seem to have higher odds of being vaccinated when compared to those with other comorbidities, in particular kidney and liver disease. These findings raise particular concern, because it is clearly demonstrated that patients with chronic conditions, not only cardiovascular or respiratory diseases, are at higher risk of many vaccine-preventable diseases (including influenza), which increases the risk of serious complications, hospitalization, and death. Moreover, vaccine-preventable diseases are more difficult to manage because of increased risk of drug interactions and potential adverse effects [27].

In our study, flu vaccination was positively associated with being a healthcare worker compared to other work categories. Nevertheless, an extremely low percentage of healthcare workers respondents, accounting for 35.7%, received a flu shot in the last season. Although the percentage reported in our study is surprisingly higher compared to that reported in the literature in Italian healthcare workers, ranging from 14% [28] to 16% [29], it underlines the need for urgent measures to increase vaccination coverage in Italian healthcare workers. The vaccination of healthcare workers against influenza is a key component of infection control, and the rationale for it is based on the need to protect the healthcare workers and their high-risk patients in nursing homes, hospitals, and outpatient clinics. Moreover, in this time of extreme demand, reducing the absenteeism due to influenza and its complications contributes to the preservation of the essential healthcare services.

The finding that nursing home residents aged 65 years or older have 61% higher odds of not being vaccinated compared to the rest of the population was unexpected and definitively worrisome. The overall flu shot rate in 221 residents in nursing home aged 65+ was similar (53.4%) to those of the general population and EPICOVID19 participants (Supplementary Table S2). However, when considering their age distribution (mean age 85.0 ± 8.3), the rate was remarkably lower than in EPICOVID19 non-resident peers (mean age 70.8 ± 5.5, rates shown in Supplementary Table S1), retired peers (mean age 69.6 ± 6.3), and younger residents (vaccination rate = 76.4%). These subjects have high rates of underlying medical conditions, and therefore, they are at higher risk of infectious disease outbreaks. As we have learned during the recent pandemic, about half of deaths due to COVID-19 were among nursing homes residents, underlying a critical health disparity that needs to be addressed urgently. Increased vaccination rates and increased testing for preventable infectious disease of residents and staff, and increased access to personal protective measures and equipment are mandatory in all nursing homes in order to protect this vulnerable subgroup of our population [30,31,32].

### Strengths and Limitations

The main strength of our research was the possibility of assessing factors associated with flu vaccination in a large unselected group of respondents to an online survey. In fact, Italian estimates on past flu vaccination seasons reported aggregated data for the general population and for those aged 65 years or older but failed to address among the target group which categories had significant barriers to achieve the pre-planned goal for flu vaccine coverage.

Our study has several limitations. First, the absence of subjects aged below 18 years makes it impossible to compare our data with that of the Italian general population reported by the Ministry of Health. Second, the high percentage of high education and healthcare workers observed in our sample should be considered in the interpretation of the results for the possible impact on behaviors and opportunities toward a better health. In addition, we were not able to assess the impact of the economic status on flu vaccination uptake, although the education level was used as a proxy of the economic status in the adjusted models. Third, caution is warranted in the interpretation of flu vaccination coverage of nursing home residents due to the possibility of recalling bias. Moreover, the possible presence of recall bias could not definitely be excluded also for the other categories. In the end, the sample cannot be considered fully representative of the Italian demographic curve, and consequently, our findings could be not completely generalizable to the Italian population.

## 5. Conclusions

Our data confirm a low vaccination coverage in the overall respondents to the online EPICOVID 19 survey, and in particular, it is far below the national recommendations in target populations, such as those 65 years or older, in subjects at high risk due to chronic conditions and in healthcare workers. In the perspective of the next fall winter season, with the probable co-circulation of influenza and SARS-CoV-2 in the northern hemisphere, the population will remain vulnerable to concurrent epidemics. The morbidity and mortality impact will be directly related to the strength of the public health response, which must be based on effective infection prevention tools currently available: the non-pharmacologic interventions (i.e., social distancing and face masks) and the widespread implementation of influenza vaccination to fill the gap of missed vaccination opportunities reported in the past flu seasons.

## Figures and Tables

**Table 1:** Characteristics of study participants according to being vaccinated for influenza or not in the 2019/2020 flu season.

Characteristics		Flu Shot during 2019/2020 Flu Season		
		No N = 157,004 (79.0)	Yes N = 41,818 (21.0)	Total N = 198,822 (100)
Sex at birth	Females	95,402 (80.4)	23,255 (19.6)	11,8657 (59.7)
	Males	61,602 (76.8)	18,563 (23.2)	80,165 (40.3)
Class of age	<65 years	14,3921 (84.3)	26,809 (15.7)	170,730 (85.9)
	65+ years	13,083 (46.6)	15009 (53.4)	28,092 (14.1)
Age		45.9 ± 13.6	55.8 ± 15.9	48.0 ± 14.7
Educational level	Low	8662 (73.1)	3192 (26.9)	11,854 (6.0)
	Middle	56,058 (81.5)	12742 (18.5)	68,800 (34.6)
	High	92,284 (78.1)	25884 (21.9)	118,168 (59.4)
Employment status	Employed	114,488 (83.7)	22368 (16.3)	136,856 (68.8)
	Student	11,781 (87.7)	1655 (12.3)	13,436 (6.8)
	Unemployed	8064 (87.8)	1120 (12.2)	9184 (4.6)
	Retired	14,056 (49.7)	14252 (50.3)	28308 (14.2)
	Other	8615 (78.0)	2423 (22.0)	11,038 (5.6)
Morbidities or clinical conditions	Lung diseases	7497 (65.4)	3959 (34.6)	11,456 (5.8)
	Heart disease	2996 (47.5)	3318 (52.5)	6314 (3.2)
	Metabolic disorders	5662 (60.7)	3663 (39.3)	9325 (4.7)
	Renal diseases	1044 (61.8)	644 (38.2)	1688 (0.8)
	Oncological diseases	3809 (60.0)	2541 (40.0)	6350 (3.2)
	Diseases of the immune system	12,998 (74.2)	4516 (25.8)	17,514 (8.8)
	Liver diseases	999 (67.2)	488 (32.8)	1487 (0.7)
	Surgical procedures	5499 (72.3)	2103 (27.7)	7602 (3.8)
Number of morbidities	None	123,240 (82.6)	25,928 (17.4)	149,168 (75.0)
	One	28,077 (70.6)	11,689 (29.4)	39,766 (20.0)
	Two	4782 (59.2)	3297 (40.8)	8079 (4.1)
	Three or more	905 (50.0)	904 (50.0)	1809 (0.9)
Work categories recommended for flu vaccine	Health professionals	9474 (64.3)	5262 (35.7)	14,736 (7.4)
	Armed forces	1610 (79.8)	407 (20.2)	2017 (1.0)
	Agricultural, forestry, and fishery workers	674 (82.3)	145 (17.7)	819 (0.4)
Other conditions for recommending flu vaccine	Pregnancy	2446 (76.7)	743 (23.3)	3189 (1.6)
	Residents in nursing home	129 (39.0)	202 (61.0)	331 (0.2)
	<65 years	26 (23.6)	84 (76.4)	110 (0.06)
	65+ years	103 (46.6)	118 (53.4)	221 (0.11)
	Cohabitants at risk	26,883 (70.5)	11,225 (29.5)	38,108 (19.2)
	Not self-sufficient in carrying out daily activities	425 (46.0)	499 (54.0)	924 (0.5)
Self-perceived health status	Good	13,5325 (80.9)	31942 (19.1)	16,7267 (84.1)
	Adequate	20,585 (69.2)	9159 (30.8)	29744 (15.0)
	Bad	1094 (60.4)	717 (39.6)	1811 (0.9)

**Table 2:** Multivariable binary logistic regression analysis on the probability of being vaccinated during the 2019/2020 flu season or not according to being 18–64 years old or ≥ 65 years old.

Characteristics	Age 18–64 years N = 26,809/170,730 (15.7)		Age ≥ 65 years N = 15,009/28,092 (53.4)	
	aOR (95%CI)	*p*	aOR (95%CI)	*p*
Sex and pregnancy	-	-	-	-
Males	1	-	1	-
Not pregnant females	0.83 (0.81 to 0.85)	0.000	1.19 (1.13 to 1.25)	0.000
Pregnant females	1.68 (1.54 to 1.85)	0.000		
Age, *per 10 years more*	1.24 (1.22 to 1.26)	0.000	2.01 (1.90 to 2.12)	0.000
Educational level	-	-	-	-
Low	1	-	1	-
Middle	1.08 (1.01 to 1.16)	0.034	1.06 (0.98 to 1.16)	0.134
High	1.59 (1.49 to 1.71)	0.000	1.40 (1.29 to 1.52)	0.000
Employment status	-	-	-	-
Employed	1	-	1	-
Student	1.50 (1.40 to 1.59)	0.000	-	-
Unemployed	0.88 (0.82 to 0.94)	0.000	1.11 (0.80 to 1.54)	0.532
Retired	1.65 (1.54 to 1.76)	0.000	1.52 (1.40 to 1.65)	0.000
Other	1.09 (1.02 to 1.15)	0.006	1.24 (1.10 to 1.40)	0.001
Morbidities or clinical conditions	-	-	-	-
Lung diseases	1.95 (1.85 to 2.05)	0.000	1.91 (1.73 to 2.12)	0.000
Heart disease	2.15 (1.98 to 2.33)	0.000	1.54 (1.42 to 1.67)	0.000
Metabolic disorders	1.68 (1.58 to 1.78)	0.000	1.33 (1.23 to 1.44)	0.000
Renal diseases	1.41 (1.22 to 1.63)	0.000	1.10 (0.91 to 1.33)	0.305
Oncological diseases	1.62 (1.50 to 1.75)	0.000	1.16 (1.06 to 1.27)	0.001
Diseases of the immune system	1.17 (1.12 to 1.23)	0.000	1.02 (0.94 to 1.11)	0.583
Liver diseases	1.00 (0.85 to 1.18)	0.972	0.92 (0.76 to 1.12)	0.410
Surgical procedures	1.06 (0.99 to 1.14)	0.093	1.13 (1.02 to 1.26)	0.021
Work categories recommended for flu vaccine	-	-	-	-
Not recommended	1	-	1	-
Agricultural, forestry, and fishery workers	0.79 (0.62 to 1.00)	0.047	0.97 (0.66 to 1.44)	0.895
Armed forces	0.97 (0.85 to 1.12)	0.699	1.24 (0.93 to 1.64)	0.139
Health professionals	2.99 (2.87 to 3.11)	0.000	1.54 (1.36 to 1.74)	0.000
Other conditions for recommending flu vaccine	-	-	-	-
Residents in nursing home	7.20 (4.29 to 12.10)	0.000	0.39 (0.28 to 0.54)	0.000
Presence of persons at risk in the household	1.36 (1.31 to 1.40)	0.000	1.42 (1.35 to 1.50)	0.000
Not self-sufficient in carrying out daily activities	1.83 (1.41 to 2.38)	0.000	0.54 (0.43 to 0.69)	0.000
Self-perceived health status	-	-	-	-
Good	1	-	1	-
Adequate	1.15 (1.10 to 1.19)	0.000	1.16 (1.10 to 1.23)	0.000
Bad	1.24 (1.08 to 1.42)	0.002	1.36 (1.12 to 1.67)	0.002

## Supplementary Materials

The following are available online at https://www.mdpi.com/2076-393X/8/4/669/s1. Supplementary Table S1. Vaccination coverage in participants according to the class of age. Supplementary Table S2. Vaccination coverage per class of age according to the Italian official national data for the 2019/2020 flu season and in the EPICOVID19 respondents. Supplementary Table S3. Multivariable binary logistic regression analysis on the probability of being or not vaccinated during the 2019/2020 flu season according to the number of reported morbidities.
