# Supplementary material for: Missed Opportunities of Flu Vaccination in Italian Target Categories: Insights from the Online EPICOVID 19 Survey
Source: Vaccines (Basel). 2020 Nov 9;8(4):669. doi: 10.3390/vaccines8040669 (PMC7712748; doi:10.3390/vaccines8040669)
Supplement: Supplementary file 1 [file vaccines-08-00669-s001.pdf]

Class of age	Flu shot during 2019/2020 flu season<br>No<br>N=157004 (79.0)	Yes<br>N=41818 (21.0)	Total<br>N=198822 (100)
18-24	8916 (89.0)	1099 (11.0)	10,015 (5.0)
25-29	12,965 (87.6)	1839 (12.4)	14,804 (7.4)
30-34	15,539 (87.1)	2311 (12.9)	17,850 (9.0)
35-39	17,049 (85.6)	2864 (14.4)	19,913 (10.0)
40-44	17,260 (85.0)	3036 (15.0)	20,296 (10.2)
45-49	19,391 (86.4)	3055 (13.6)	22,446 (11.3)
50-54	19,848 (86.0)	3235 (14.0)	23,083 (11.6)
55-59	18,753 (81.7)	4195 (18.3)	22,948 (11.5)
60-64	14,200 (73.3)	5175 (26.7)	19,375 (9.7)
65-69	7885 (55.3)	6373 (44.7)	14,258 (7.2)
70-74	3504 (41.1)	5018 (58.9)	8522 (4.3)
75-79	1081 (35.1)	2003 (64.9)	3084 (1.6)
80-84	379 (27.5)	998 (72.5)	1377 (0.7)
85-89	136 (27.0)	367 (73.0)	503 (0.3)
90+	98 (28.2)	250 (71.8)	348 (0.2)

Supplementary Table 1. Vaccination coverage in participants according to the class of age.

		Flu shot rates by class of age	
		**National 2109-2020**	**EPICOVID19**
Class of age	18-44 years	574,335/18,526,934 (3.1)	11,149/82,878 (13.5)
	45-64 years	1,749,887/18,227,994 (9.6)	15,660/87,852 (17.8)
	18-64 years	2,324,222/36,754,928 (6.3)	26,809/170,730 (15.7)
	65+ years	7,615,037/13,946,954 (54.6)	15,009/28,092 (53.4)
	60-64 years	-	5,175/19,375 (26.7)

Supplementary Table 2. Vaccination coverage per class of age according to the Italian official national data and in the EPICOVID19 respondents.

	**Age 18-64 years N=26,809/170,730 (15.7)**		**Age ≥ 65 years N=15,009/28,092 (53.4)**	
	**aOR (95%CI)**	***p***	**aOR (95%CI)**	***p***
Number of morbidities				
None	1		1	
One	1.54 (1.49 to 1.59)	0.000	1.43 (1.35 to 1.51)	0.000
Two	2.18 (2.05 to 2.33)	0.000	1.70 (1.55 to 1.86)	0.000
Three or more	2.60 (2.26 to 2.99)	0.000	1.66 (1.42 to 1.94)	0.000

Supplementary Table 3. Multivariable binary logistic regression analysis on the probability of being or not vaccinated during the 2019/2020 flu season according to the number of reported morbidities.
